# Feeding Bottles Usage and the Prevalence of Childhood Allergy and Asthma

**DOI:** 10.1155/2012/158248

**Published:** 2012-01-15

**Authors:** Nai-Yun Hsu, Pei-Chih Wu, Carl-Gustaf Bornehag, Jan Sundell, Huey-Jen Su

**Affiliations:** ^1^Department of Environmental and Occupational Health, College of Medicine, National Cheng Kung University, Tainan 70403, Taiwan; ^2^Department of Occupational Safety and Health, Chang Jung Christian University, Tainan 71101, Taiwan; ^3^Public Health Sciences, Karlstad University, 651 88 Karlstad, Sweden; ^4^International Centre for Indoor Environment and Energy, Technical University of Denmark, 2800 Lyngby, Denmark

## Abstract

This study aimed to examine the association between the length of use of feeding bottles or pacifiers during childhood and the prevalence of respiratory and allergic morbidities. A large-scale questionnaire survey was performed in day care centers and kindergartens (with children's ages ranging from 2 to 7 years) in southern Taiwan, and a total of 14,862 questionnaires completed by parents were finally recruited for data analysis. Effects of using feeding bottles on children's wheezing/asthma (adjusted OR: 1.05, 95% CI 1.00–1.09), allergic rhinitis (adjusted OR: 1.04, 95% CI 1.00–1.08), and eczema (adjusted OR: 1.07, 95% CI 1.01–1.2) were found. Moreover, significant dose-dependent relationships were further established after an adjustment for confounders was performed that included children's ages, gender, gestational age, birth weight, length of breastfeeding, the age when first given infant formula or complementary foods, family history, parental educational levels, and smoking status, as well as the problem of indoor water damage. This study was the first to reveal the potential risk of using plastic consumer products such as feeding bottles on the reported health status of preschool children in Asian countries.

## 1. Introduction

Because of the multifactorial nature of pathogenesis, it is much clearer now that the rising prevalence and morbidity of childhood asthma and allergic diseases cannot be explained only by genetics and allergen exposure. Several chemicals from many common consumer products have been shown to have toxicity in animal studies and have also been suggested to have an impact on human health. For example, bisphenol A (BPA) is used to manufacture polycarbonate plastic and epoxy resins, which are used in a large number of products found indoors, such as epoxy, building components, and electronic equipment as well as protective coatings on food containers and baby bottles. Toxicological studies of animals have suggested that exposure to BPA is associated with morphologic, functional, and behavioural anomalies related to reproduction. Phthalate esters are stabilizers and plasticizers in commonly used consumer products [1] such as personal care products, food packaging, medical equipment, toys, and building materials. Experimental studies during the past decade have proposed their role as an adjuvant on T_H_2 differentiation or as having an association with the early phases of inflammatory response [2, 3].

We are commonly exposed to various chemicals with potential health concerns in our daily lives through the use of consumer products; however, there have been no studies attempting to verify whether the use of these kinds of products is associated with health status, especially in the case of the most susceptible group, young children. The current analysis was aimed at an examination of the association between the length of use of pacifiers or feeding bottles during childhood and the prevalence of respiratory and allergic disease/symptoms in a Taiwanese population.

## 2. Materials and Methods

### 2.1. Study Subjects

In 2005 and 2006, randomly selected kindergartens (*n* = 201) and day care centres (*n* = 259) in the Greater Tainan metropolitan area of southern Taiwan were asked through telephone interviews to participate in a questionnaire survey aimed at identifying the relationship between indoor environmental quality in the home to children's health. An average of 73% of the successfully contacted schools (*n* = 335) agreed to participate and to help send a questionnaire to the parents of children between the ages of 2 and 6 who attended their schools. A total of 14,862 questionnaires were returned with a 68% response rate of questionnaires sent to the 355 kindergartens and day care centres. The study was approved by the Human Experiment and Ethics Committee at National Cheng Kung University Hospital in Tainan, Taiwan.

### 2.2. Questionnaire

Questions for assessment of the children's asthma and allergy were adopted from the International Study of Asthma and Allergies in Childhood (ISAAC) protocol [4], including the following questions.

Core question for wheezing and asthma.

- Has your child *ever* had wheezing or whistling in the chest at any time in the past?
- *In the last 12 months*, has your child had a dry cough at night for more than two weeks, apart from a cough associated with a cold or chest infection?
- Has your child been diagnosed with asthma by a doctor?

Core question for allergic rhinitis.

- In the past 12 months, has your child had a problem with sneezing, or a runny, or a blocked nose when he/she DID NOT have a cold or the flu?
- Has your child been diagnosed with hay fever or allergic rhinitis by a doctor?

Core question for eczema.

- Has your child ever had an itchy rash (eczema), which was coming and going for the last 6 months?

For the environmental condition component used on the questionnaire for this study, questions that were identical to the worldwide Dampness in Buildings and Health (DBH) study [5] were adopted. Questions regard to the length of use of pacifiers or feeding bottles in the survey were as follows.

- Did your child use a pacifier? If yes, at what age did your child stop using it?
- Did your child use a feeding bottle? If yes, at what age did your child stop using feeding bottles?

There were eight options for answering either question, including “(1) never used, (2) stopped use before 1 year old, (3) stopped use before 2 years old, (4) stopped use before 3 years old, (5) stopped use before 4 years old, (6) stopped use before 5 years old, (7) stopped use before 6 years old, and (8) is still using.”

### 2.3. Data Analysis

Differences in the percentages between any of the groups shown in Table 2 were calculated using a chi-square, while the *P* value for trends was applied using a chi-square for the trend test (ordinary by ordinary) for ordinal data. Multivariable logistic regression was applied to examine the effect after adjusting for potential confounders. All statistical analysis was performed with the SPSS, version 17 (Chicago, IL, USA).

## 3. Results

Questionnaires were mostly filled out by mothers (62.3%).  Table 1 presents the characteristics of 14,862 children, including their ages, gender, gestational age, birth weight, length of being breastfed, the age first given infant formula or complementary foods, family history, parental educational levels, and smoking status. Moreover, a high prevalence of reported water damage in the home (35.7%) was shown in southern Taiwan.

The lifetime prevalence of parental reporting of wheezing/asthma, allergic rhinitis, and eczema among preschool children is tabulated in Table 2. The average prevalence of doctor-diagnosed asthma, doctor-diagnosed allergic rhinitis and the reporting of eczema symptoms during the 6 months prior to this study among preschool children in Taiwan was 9.3%, 19.3%, and 17.6%, respectively. More than half of the children studied (50.8%) had rhinitis symptoms, including sneezing or a runny or blocked nose when they were absent resulting from having had a cold or a flu in the previous 12 months. The highest rate of diagnosed asthma was found at the age of 5 years old, at 10.2%. Moreover, it was apparent that the prevalence of allergic rhinitis and reported symptoms was increasing along with the age of children, whereas an inverse situation was found for eczema. As to the morbidities of wheezing/asthma and rhinitis, children with any one of related symptoms or diseases were recognized as cases. Overall, there were 34.9% and 53.6% of preschool children with reported morbidities of wheezing/asthma and allergic rhinitis, respectively, in Taiwan.

With regard to clinical data, physician-diagnosed health statuses of young children, especially in the case of asthma, were not stable and permanent until the age of 3 years. The current analysis therefore excluded subjects who were younger than 3 years old (*n* = 489, Table 1) and those missing age information (*n* = 1700, Table 1). The length of using pacifiers or feeding bottles among the study children was stratified into a quartile range as shown in Table 3. A total of 24.3% children never had used feeding bottles or had used them until they were 2 years old; 25.3% children had stopped use between 2 and 3 years old; 24.2% children had stopped use between 3 and 5 years old; the remaining 26.2% of the children had used these items until the time of this investigation. Results revealed that the prevalence rates of wheezing/asthma, allergic rhinitis, and eczema in the four groups were increasing significantly (*P* value for trend <0.05), with higher quartiles representing a longer length of using feeding bottles among the children who were subjects in this study. The only statistically significant trend between outcomes and the length of using pacifiers was found for the reported symptom of allergic rhinitis (*P* value for trend = 0.025).

The relationship between the length of use of feeding bottles and the prevalence of disease was adjusted for all confounding factors shown in Table 4. Significant effects of using feeding bottles on children's wheezing/asthma (adjusted OR: 1.05, 95% CI 1.00–1.09), allergic rhinitis (adjusted OR: 1.04, 95% CI 1.00–1.08), and eczema (adjusted OR: 1.07, 95% CI 1.01–1.12) were found. The significant dose-dependent effects (*P* value for trend <0.05) between higher quartiles and the risk for having diseases or symptoms remained even after the adjustment for confounders was performed. Children who had used the feeding bottle until the time of this study (higher than the 75th percentile) were associated with a significant risk for reporting outcomes of interest compared to the first quartile (less than the 25th percentile) of subjects who had never used or stopped use before 2 years old.

## 4. Discussion

This study was the first to reveal that the use of feeding bottles among children might be one of the risk factors for the development of asthma and allergic diseases in Asian countries. Overall, we observed that a longer period of use of feeding bottles indicated a higher risk of diseases/symptoms among preschool children after adjustment for various confounders, including the children's age, gender, gestational age, birth weight, length of time being breastfed, the age first given infant formula or complementary foods, family history, parental educational levels, and smoking status, as well as the problem of indoor water damage.

Rising prevalence and morbidity of childhood asthma and allergic diseases has been observed globally [6, 7]. Taiwan has also been facing the same challenges during the past 20 years [8–10]. Previous studies have reported that about 80–90% of patients first succumb to allergic diseases before they are 5 years old [11]. However, none of the studies on this topic has investigated the prevalence of diseases among preschool-aged children in Taiwan. This study was the first to conduct a regional survey of children with an age range between 2 and 6 years old in order to explore the potential risk factors contributing to the development or presence of asthma and allergic diseases. From the current analysis, a prevalence of eczema was found to be the highest in children younger than 3 years old and to decrease gradually as age increased. On the contrary, the most prevalent period for allergic rhinitis was at 6 to 7 years old, while for diagnosed asthma, it was at 5 years of age. The current profile of prevalence for asthma and allergic morbidity corresponded to the theory of “atopic march,” used for describing the phenomenon of the progression of allergic disorders among predisposed children. Eczema (atopic dermatitis) is thought to be an “entry point” for subsequent allergic diseases, including asthma and allergic rhinitis [12, 13].

The issue of plastic and health has attracted enormous attention in recent years [14], and there is also a possibility that any harmful chemicals emitted from pacifiers or feeding bottles could be the causal factor associated with this relationship. Only limited literature has reported relationships between childhood allergic diseases and the use of feeding bottles, pacifiers, or toys. One study from Japan indicated that the presence of asthmatic symptoms and eczema was associated with the use of latex for newborns who were less than 1 year old [15]. Another study conducted in Pakistan has shown early bottle feeding to be associated with higher total serum IgE levels in the study children [16]. Morass et al. in Austria also reported that children who had used pacifiers exhibited a higher percentage of wheezing symptoms during the previous 12 months [17]. The most interesting point is that a positive dose-dependent relationship was established by Morass et al. [17] between the frequency of boiling pacifiers and the percentage of children with wheezing or asthma. The authors tended to explain these phenomena through the “hygiene hypothesis,” since boiling the pacifier less frequently might be a measure of generally lower hygiene levels, whereas boiling the pacifier daily might result in a decline in children's microbial exposure and, therefore, to increases risk of developing asthma and allergic diseases [17]. However, a study in China found that BPA was released within 24 hours from four brands of baby bottles at room temperatures of 24°C, 40°C, and 100°C, while increased temperatures led to higher release of BPA from the baby bottles [18]. Kubwabo et al. also showed the level of BPA from polycarbonate (PC) bottles increased with temperature and incubation time [19]. BPA has been concluded to might enhance allergic sensitization and bronchial inflammation during perinatal exposure and responsiveness in a susceptible animal model of asthma [20, 21]. A likely potential health risk of plastic exposure through the use of feeding bottles on asthma/allergies is therefore highly speculated. On the other hand, Sugita et al. [22] reported high levels of di-2-ethylhexyl phthalate (DEHP) (average 162 mg/g, 2.0–380 mg/g) in pacifiers and other related products that were used frequently by infants. Exposure to phthalates, one of most common plasticizers used in daily life, has shown its potential to be correlated with allergies and asthma in both animal and epidemiologic studies [2, 3]. Our recent publication also revealed that levels of indoor dust-borne benzylbutyl phthalate (BBzP) and dibutyl phthalate (DBP) as well as the urinary metabolites mono-n-butyl phthalate (MBP) and mono-2-ethylhexyl phthalate (MEHP) are associated with increased risks of allergies and asthma after taking into account exposure to other indoor pollutants [23].

We understand that the evidence might not be strong enough, constrained by the nature of a cross-sectional study design, and the casual relationship could not be established. However, it is also evident that such a study is aimed to raise new hypotheses between emergent exposures and the outcomes of significant interest. After further adjustments of confounders, it is believed that potential health concern of using feeding bottles should be attended to in the future.

## 5. Conclusions

While people have recently had dramatically increased exposure to various emerging chemicals in large amounts, the group about which there is the most concern has been children, and the current study was the first to reveal the potential risk of using plastic consumer products, such as feeding bottles, as it was indicated from reported health status in an East Asian population. The specific underlying mechanism of feeding bottles usage resulting in the observed health outcomes warrants future investigation.

## Figures and Tables

**Table 1:** Characteristics of study children.

	*n* (%)	*n* (%) of missing data*
*Questionnaire filled out by*		*1,030 *(*6.9*)
Both father and mother	3,022 (21.8)	
Mother only	8,619 (62.3)	
Father only	1,738 (12.6)	
Grandparents	216 (1.6)	
Others	237 (1.7)	
*Age*		*1,700 *(*11.4*)
Less than 3 years old	489 (3.7)	
3 years old	1,631 (12.4)	
4 years old	3,740 (28.4)	
5 years old	5,021 (38.1)	
6 ~ 7 years old	2,281 (17.4)	
*Gender*		*2,278 *(*15.3*)
Female	6,101 (48.5)	
Male	6,483 (51.5)	
*Gestational age*		*521 *(*3.5*)
Before week 32	205 (1.4)	
In week 32–36	2,178 (15.2)	
In week 37–42	10,731 (74.8)	
In week 43 or later	745 (5.2)	
Unknown	482 (3.4)	
*Birth weight*		*230 *(*1.5*)
Less than 2500 grams	932 (6.4)	
2500–4200 grams	13,376 (91.4)	
More than 4200 grams	180 (1.2)	
Unknown	144 (1)	
*Breastfed totally or partly until*		*279 *(*1.9*)
Never	5,630 (38.6)	
Younger than 3 months	6,087 (41.7)	
3–6 months	1,371 (9.4)	
Older than 6 months	1,495 (10.3)	
*The age first given infant formula*		*969 *(*6.5*)
Never	248 (1.8)	
Younger than 3 months	9,739 (70.1)	
3–6 months	2,568 (18.5)	
Older than 6 months	1,338 (9.6)	
*The age first introducing complementary foods*		*590 *(*4*)
Never	111 (0.8)	
Younger than 3 months	220 (1.5)	
3–6 months	5,761 (40.4)	
Older than 6 months	8,180 (57.3)	
*Ever had allergic symptoms to foods*		*449 *(*3*)
Unknown	1,022 (7.1)	
Never	11,947 (82.9)	
Yes, ever had allergic reaction to	1,444 (10.0)	
seafood	768 (53.1)	
milk or dairy products	269 (18.4)	
eggs	233 (16.1)	
peanuts	136 (9.4)	
fish	124 (8.6)	
fruit	101 (7.0)	
soya, peas, beans	48 (3.3)	
vegetables	44 (3.1)	
nuts, almond	43 (3)	
flour	34 (2.4)	
others	376 (26.1)	
*Family history*		*424 *(*2.9*)
Paternal asthma	310 (2.1)	
Paternal allergic rhinitis or eczema	2,709 (18.8)	
Maternal asthma	408 (2.8)	
Maternal allergic rhinitis or eczema	2,733 (18.9)	
Sibling with asthma	643 (4.4)	
Sibling with allergic rhinitis or eczema	2,361 (16.4)	
*Paternal educational levels*		*336 *(*2.3*)
Junior and junior high school	2,348 (16.1)	
Senior high school	5,943 (40.9)	
Undergraduate degree	5,216 (35.9)	
Graduate degree	1,019 (7)	
*Maternal educational levels*		*369 *(*2.5*)
Lower than junior high school	2,117 (14.6)	
Senior high school	6,510 (44.9)	
Undergraduate degree	5,476 (37.8)	
Graduate degree	390 (2.7)	
*Parents smoked during the child's first year of life*		*627 *(*4.9*)
Either father or mother smoked	6,303 (49.7)	
*Indoor problems with water damage*		*215 *(*1.7*)
In any room of the home	3,205 (35.7)	

*The percentage of missing data among 14862 subjects.

**Table 2:** Prevalence of diseases or symptoms among study children.

	Total population, % (*n*)	Stratified by the age of child while questionnaire survey, % (*n*)					
		Less than 3 years old	3 years old	4 years old	5 years old	6 ~ 7 years old	*P* value*
Wheezing/asthma							
Wheezing ever	28.6 (4090)	33.9 (161)	31.8 (507)	28.3 (1020)	27.6 (1325)	28.3 (618)	**0.002**
Cough at night last 12 months	11.2 (1611)	11.3 (54)	12.5 (198)	11.1 (404)	10.7 (518)	10.5 (232)	0.326
Doctor-diagnosed asthma	9.3 (1176)	6.2 (27)	8.6 (120)	8.7 (282)	10.2 (437)	9.6 (184)	**0.021**
*Any one of the abovementioned *	*34.9 *(*5146*)	*39.0 *(*190*)	*38.3 *(*623*)	*34.5 *(*1283*)	*33.8 *(*1685*)	*33.9 *(*766*)	***0.002***
Allergic rhinitis							
Rhinitis last 12 months	50.8 (7301)	44.2 (212)	49.5 (786)	49.1 (1778)	51.7 (2517)	53.9 (1187)	**<0.001**
Doctor-diagnosed rhinitis	19.3 (2791)	12.6 (60)	16.6 (265)	19.6 (716)	20.3 (992)	21.2 (470)	**<0.001**
*Any one of the abovementioned *	*53.6 *(*7906*)	*46.9 *(*229*)	*51.9 *(*843*)	*52.6 *(*1957*)	*54.4 *(*2714*)	*56.7 *(*1280*)	***<0.001***
Eczema							
Eczema during last 6 months	17.6 (2539)	29.2 (140)	19.9 (318)	17.4 (635)	16.4 (798)	16.3 (361)	**<0.001**

*P values were calculated by Pearson Chi-Square to compare the difference of percentages among five age groups.

**Table 3:** The association between the length of using feeding bottles or pacifiers and childhood allergic and respiratory morbidities.

% (*n*)	Quartile of using length for feeding bottles or pacifiers				*P* value**^†^**	*P* value for trend^‡^
	<25th percentile	25th–50th	50th–75th	>75th percentile		
Length of using feeding bottle						
*Range of quartile *	*Never used and stopped use before 2* *yrs, 24.3 *(*2979*)	*Stopped use between 2 and 3* *yrs, 25.3 *(*3103*)	*Stopped use between 3 and 5* *yrs, 24.2 *(*2960*)	*Used until now, 26.2 *(*3205*)		
Wheezing/asthma	33.0 (976)	34.8 (1076)	34.2 (1007)	37.5 (1199)	**0.002**	**0.001**
Allergic rhinitis	52.3 (1550)	54.4 (1680)	54.1 (1594)	55.4 (1768)	0.102	**0.025**
Eczema	15.6 (452)	17.1 (516)	16.1 (467)	19.7 (620)	**<0.001**	**<0.001**
Length of using pacifiers						
*Range of quartile *	*Never used, 27.7 *(*3433*)	*Stopped use before 1* *yr, 27.0 *(*3345*)	*Stopped use between 1 and 2* *yrs, 25.5 *(*3162*)	*Stopped use between 2 and 6* *yrs as well as used until now, 19.9 *(*2465*)		
Wheezing/asthma	33.0 (1129)	36.1 (1202)	35.1 (1104)	35.4 (869)	**0.050**	0.084
Allergic rhinitis	51.6 (1764)	55.2 (1834)	55.4 (1745)	54.1 (1328)	**0.007**	**0.025**
Eczema	16.5 (555)	16.9 (550)	17.3 (537)	17.8 (428)	0.636	0.194

^†^ *P* values were calculated by Pearson Chi-Square to compare the difference of percentages among four quartile groups.

^‡^ *P* values for trend were calculated by Chi-Square of Ordinal by Ordinal to examine the trend of correlation between disease rates and length of use.

**Table 4:** The dose-effect relationship between disease prevalence and the age of stopping use of feeding bottles or pacifiers.

	Crude OR (95% CI)^†^	Adjusted OR (95% CI)^‡^	Quartile of using length for feeding bottles or pacifiers, adjusted OR (95% CI)^‡^				*P* values for trend*
			<25th (Ref.)	25th–50th	50th–75th	>75th	
Length of using feeding bottle							
Wheezing or asthma	**1.06 (1.03–1.10)**	**1.05 (1.00–1.09)**	1.00	1.10 (0.96–1.25)	1.11 (0.97–1.27)	**1.16 (1.01–1.32)**	**0.035**
Allergic rhinitis	**1.04 (1.00–1.07)**	**1.04 (1.00–1.08)**	1.00	1.09 (0.96–1.23)	1.01 (0.89–1.14)	**1.18 (1.03–1.34)**	**0.052**
Eczema	**1.09 (1.04–1.13)**	**1.07 (1.01–1.12)**	1.00	1.08 (0.91–1.28)	1.06 (0.89–1.25)	**1.25 (1.06–1.48)**	**0.017**
Length of using pacifiers							
Wheezing or asthma	**1.03 (1.00–1.07)**	1.02 (0.98–1.06)	1.00	1.11 (0.98–1.26)	1.08 (0.95–1.22)	1.07 (0.94–1.23)	0.370
Allergic rhinitis	**1.04 (1.00–1.07)**	1.02 (0.98–1.06)	1.00	1.08 (0.96–1.22)	1.11 (0.98–1.25)	1.04 (0.91–1.18)	0.441
Eczema	1.03 (0.99–1.08)	1.03 (0.98–1.09)	1.00	1.05 (0.90–1.23)	1.00 (0.85–1.17)	1.14 (0.96–1.35)	0.259

^†^Crude univariable effects were calculated by logistic regression.

^‡^ORs were calculated using multiple logistic regression with the adjustment of all factors tabulated in Table 1, including the persons completing the questionnaire, parental educational levels and smoking status, family history, child's gender, age, gestational age, birth weight, breastfeeding history, use of formula and complementary foods, food allergy status, and report of indoor water damage.

*P value for trend was calculated using the regression model while the predictor was considered as the continuous variable with the above-mentioned adjustment.

## References

[1] Schettler T (2006). Human exposure to phthalates via consumer products. *International Journal of Andrology*.

[2] Bornehag CG, Nanberg E (2010). Phthalate exposure and asthma in children. *International Journal of Andrology*.

[3] Kimber I, Dearman RJ (2010). An assessment of the ability of phthalates to influence immune and allergic responses. *Toxicology*.

[4] Pearce N, Weiland S, Keil U (1993). Self-reported prevalence of asthma symptoms in children in Australia, England, Germany and New-Zealand—an international comparison using the ISAAC protocol. *European Respiratory Journal*.

[5] Bornehag CG, Sundell J, Sigsgaard T (2004). Dampness in buildings and health (DBH): report from an ongoing epidemiological investigation on the association between indoor environmental factors and health effects among children in Sweden. *Indoor Air*.

[6] Pearce N, Aït-Khaled N, Beasley R (2007). Worldwide trends in the prevalence of asthma symptoms: Phase III of the International Study of Asthma and Allergies in Childhood (ISAAC). *Thorax*.

[7] Eder W, Ege MJ, Von Mutius E (2006). The asthma epidemic. *New England Journal of Medicine*.

[8] Tsuang HCA, Su HJJ, Kao FF, Shih HC (2003). Effects of changing risk factors on increasing asthma prevalence in southern Taiwan. *Paediatric and Perinatal Epidemiology*.

[9] Liao PF, Sun HL, Lu KH, Lue KH (2009). Prevalence of childhood allergic diseases in Central Taiwan over the past 15 years. *Pediatrics and Neonatology*.

[10] Lee YL, Lin YC, Hwang BF, Guo YL (2005). Changing prevalence of asthma in Taiwanese adolescents: two surveys 6 years apart. *Pediatric Allergy and Immunology*.

[11] Bisgaard H, Bonnelykke K (2010). Long-term studies of the natural history of asthma in childhood. *Journal of Allergy and Clinical Immunology*.

[12] Spergel JM, Paller AS (2003). Atopic dermatitis and the atopic march. *Journal of Allergy and Clinical Immunology*.

[13] Ker J, Hartert TV (2009). The atopic march: what's the evidence?. *Annals of Allergy, Asthma & Immunology*.

[14] Halden RU (2010). Plastics and health risks. *Annual Review of Public Health*.

[15] Kimata H (2004). Latex allergy in infants younger than 1 year. *Clinical and Experimental Allergy*.

[16] Satwani H, Rehman A, Ashraf S, Hassan A (2009). Is serum total IgE levels a good predictor of allergies in children?. *Journal of the Pakistan Medical Association*.

[17] Morass B, Kiechl-Kohlendorfer U, Horak E (2008). The impact of early lifestyle factors on wheezing and asthma in Austrian preschool children. *Acta Paediatrica, International Journal of Paediatrics*.

[18] Li X, Ying GG, Su HC, Yang XB, Wang L (2010). Simultaneous determination and assessment of 4-nonylphenol, bisphenol A and triclosan in tap water, bottled water and baby bottles. *Environment International*.

[19] Kubwabo C, Kosarac I, Stewart B, Gauthier BR, Lalonde K, Lalonde PJ (2009). Migration of bisphenol A from plastic baby bottles, baby bottle liners and reusable polycarbonate drinking bottles. *Food Additives and Contaminants. Part A*.

[20] Midoro-Horiuti T, Tiwari R, Watson CS, Goldblum RM (2010). Maternal bisphenol a exposure promotes the development of experimental asthma in mouse pups. *Environmental Health Perspectives*.

[21] Kwak ES, Just A, Whyatt R, Miller RL (2009). Phthalates, pesticides, and Bisphenol- a exposure and the development of nonoccupational asthma and allergies: how valid are the links?. *The Open Allergy Journal*.

[22] Sugita T, Hirayama K, Nino R, Ishibashi T, Yamada T (2001). Contents of phthalate in polyvinyl chloride toys. *Shokuhin Eiseigaku Zasshi*.

[23] Hsu NY, Lee CC, Wang JY Predicted risk of childhood allergy asthma, and reported symptoms using measured phthalate exposure in dust and urine.

